# A porcine model of acute rejection for cardiac transplantation

**DOI:** 10.3389/fcvm.2025.1549377

**Published:** 2025-07-18

**Authors:** Michelle Mendiola Pla, Yuting Chiang, Carolyn Glass, David C. Wendell, Devjanee Swain-Lenz, Sam Ho, Marat Fudim, Franklin H. Lee, Lillian Kang, Matthew F. Smith, Alejandro Alvarez Lobo, Kishen Mitra, Ryan T. Gross, Chunbo Wang, Muath Bishawi, Andrew Vekstein, Krish Dewan, JengWei Chen, Amy Evans, Antonio Roki, Paul Ferrell, Kristianne M. Oristian, Salvatore V. Pizzo, Jie Li, Laura P. Hale, Paul M. Lezberg, Carmelo A. Milano, Dawn E. Bowles

**Affiliations:** ^1^ Division of Cardiothoracic Surgery, Duke University Medical Center, Durham, NC, United States; ^2^ Division of Cardiovascular and Thoracic Surgery, Aurora St. Luke’s Medical Center, Milwaukee, WI, United States; ^3^ Department of Pathology, Duke University Medical Center, Durham, NC, United States; ^4^ Duke Cardiovascular Magnetic Resonance Center, Duke University Medical Center, Durham, NC, United States; ^5^ Sequencing and Genomics Technologies Core Facility, Duke University School of Medicine, Durham, NC, United States; ^6^ Department of Molecular Genetics and Microbiology, Duke University School of Medicine, Durham, NC, United States; ^7^ Gift of Hope Organ and Tissue Donor Network, Itasca, IL, United States; ^8^ Department of Cardiology, Duke University Medical Center, Durham, NC, United States; ^9^ Division of Surgical Science, Duke University Medical Center, Durham, NC, United States; ^10^ Division of Cardiothoracic Surgery, Emory University Medical Center, Atlanta, GA, United States; ^11^ Department of Surgery, National Taiwan University Hospital, Taipei, Taiwan; ^12^ Perfusion Services, Duke University Medical Center, Durham, NC, United States; ^13^ Department of Radiation Oncology, Duke University Medical Center, Durham, NC, United States; ^14^ TransMedics, Inc., Andover, MA, United States

**Keywords:** acute rejection, cardiac transplantation, machine perfusion, disease model, translational research

## Abstract

*Ex vivo* machine perfusion has been growing in utility for preserving donor organs prior to transplantation. This modality has tremendous potential for bioengineering and conditioning organs prior to transplantation using small molecule or advanced therapeutics. To safely translate potential interventions, well characterized models of disease are crucial for testing the therapeutic and possible side effects that could manifest from the interventions. Acute cellular rejection remains a significant complication in organ transplantation that affects transplant recipients with significant morbidity and mortality. This disease could potentially be mitigated with therapeutic intervention during *ex vivo* machine perfusion. A porcine animal model of acute rejection could be characterized in order to translate human biological processes with high fidelity. The Yucatan pig breed has been increasingly used in both biomedical research and xenotransplantation applications given its similarity to the human heart. A challenge with utilizing this pig breed for designing a model of acute rejection is its highly conserved ancestral lineage, which could make it difficult to induce acute rejection in a timely and consistent manner. We present a detailed characterization of a porcine model of acute rejection based on swine leukocyte antigen mismatching paired with a limited period of clinically relevant immunosuppression. The result is a robust and consistent protocol that results in fulminant acute rejection of an intra-abdominally transplanted heart.

## Introduction

Well characterized animal models that closely reflect diseases or conditions observed in humans are crucial for the safe translation of therapeutics into clinical use, integrating concepts about the pathophysiology of the disease and modern diagnostic techniques that are used in the clinical setting (1). Numerous small and large animal models have been described in the context of acute rejection (AR) following solid organ transplantation, primarily through varying degrees of major histocompatibility complex mismatching between the donor and the recipient animal (2–5). Pigs are advantageous as a large animal model given their similarity to humans in anatomical size, structure, physiology and immunology, making them preferable to murine models for translational and clinical applications (6, 7). This similarity allows for safe dosage ranges to be defined and for thorough assessment of toxicology. Additionally, pigs are the preferred animal for xenotransplantation because of this high degree of similarity to humans (6, 8, 9).

We present a detailed characterization of a porcine model of acute cellular rejection (ACR) for cardiac transplantation that integrates organ preservation using normothermic *ex vivo* machine perfusion (EVMP). We previously described successful delivery of the firefly luciferase gene to porcine hearts using normothermic EVMP on the TransMedics Organ Care System (OCS) using both adenoviral and adenoviral-associated viral vectors (10, 11). This *ex vivo* method of delivery to entire hearts can be utilized to administer both small molecule and advanced therapeutics to organs prior to transplantation that can effectively protect the transplanted organ from damage caused by the recipient's immune system (2, 12).

**Figure 1:**
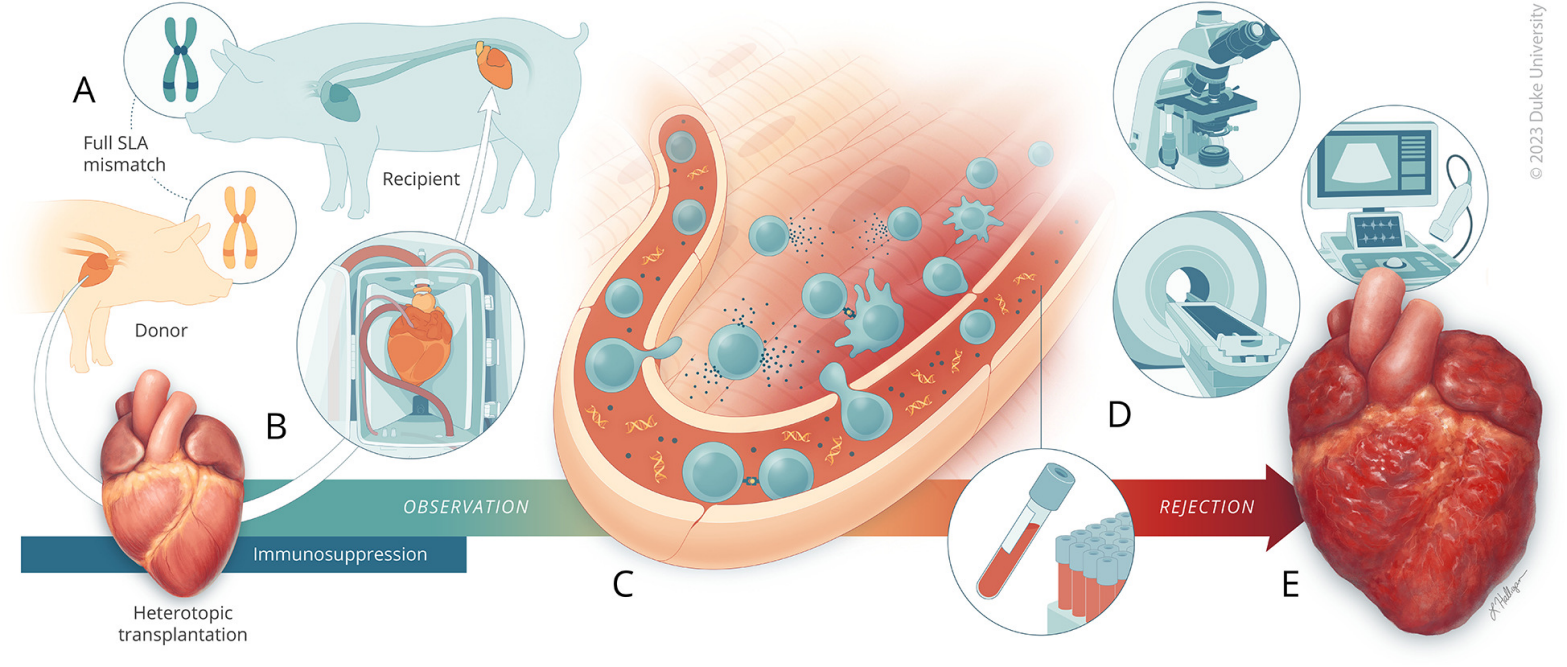
A porcine model of acute rejection for cardiac transplantation. **(A)** Yucatan pigs are first genotyped for SLA haplotype. Donor-recipient pairs are selected where there is complete mismatch of all SLA class I and class II genes. The recipient pig begins receiving immunosuppression therapy three days prior to transplantation. **(B)** The heart is procured from the donor pig and undergoes *ex vivo* normothermic machine perfusion for two-hours. The recipient pig then undergoes abdominal heterotopic heart transplantation. Following transplantation, the recipient continues to receive immunosuppression therapy for 14-days, at which time **(C)** close observation begins for monitoring acute rejection driven primarily by cellular processes involving CD4^+^ and CD8^+^ cells. **(D)** Assessments through ultrasound, CMR, histology, blood analysis, and cfDNA quantitation were used to monitor for acute rejection. **(E)** Observation was continued until fulminant rejection was reached.

To be able to effectively investigate therapeutics, a preclinical model of ACR that is able to be translated to clinical practice is required (13). One of the primary mechanisms known to drive the process of AR is the activation of T cells that recognize non-self-antigens derived from the transplanted organ. Human leukocyte antigens (HLA) are highly antigenic and can elicit a strong immune response that can lead to AR, such that it has been described that HLA matching in human transplantation has a strong influence on transplant outcomes (14). However, purpose-bred laboratory pigs may share swine leukocyte antigens (SLA) and the time required for rejection of a transplanted organ may vary widely from weeks to years. Through the mismatch of swine leukocyte antigens (SLA), the equivalents of HLA, and controlled administration of immunosuppressive medications, we describe a protocol that achieves ACR within a consistent timeframe in Yucatan pigs. In order to characterize the progression of rejection, we used an array of traditional and advanced clinical diagnostic modalities, including endomyocardial biopsy histopathology, cardiac magnetic resonance (CMR) imaging, cell-free DNA (cfDNA) sequencing analysis, and immunophenotyping (Figure 1). The model of heart transplant rejection we developed can be used for the assessment of small molecular and advanced therapeutics for the prevention and treatment of AR during cardiac transplantation, as well as to evaluate novel biomarkers of cardiac rejection. Using this animal model, we evaluated the biomarker Pro-N-Cadherin (PNC), previously described as a marker of tissue injury, on graft myocardial tissue as a marker of AR (15).

## Methods

### Animals and swine leukocyte antigen haplotyping

This study was approved by the Duke University Institutional Animal Care and Use Committee (A122-22-06). Female Yucatan pigs (Sinclair Bio Resources, Auxvasse, MO) aged 9–10 months and weighing 30–43 kg were used. All pigs underwent SLA genotyping and blood typing prior to selection as previously described (16, 17) with modifications made to the typing primer panels to broaden the allele coverage and typing resolution. The class I genes SLA-1, SLA-2, and SLA-3, and the class II genes DRB1, DQB1, and DQA were analyzed. High-resolution, allelic SLA typing was inferred based on previous literature and reports (18–21). Donor-recipient pairs were blood-type compatible, but either partially or fully SLA class I and class II mismatched.

### Heterotopic heart transplantation and ex vivo perfusion of donor heart

The donor pig was sedated using general anesthesia and intubated for ventilation. The donor heart was procured in standard fashion prior to undergoing normothermic EVMP for 2 h using the TransMedics OCS (Andover, MA). The OCS device and perfusate solution were prepared and managed as previously described (22, 23). At the end of the perfusion period, the heart was once again arrested and removed from the device. The heart was prepared for heterotopic transplantation and transplanted into the recipient pig in an intra-abdominal position via a laparotomy; an aorto-aortic anastomosis and pulmonary artery-IVC anastomosis were performed between the allograft and the recipient as previously described (22). After the heart was reanimated, the laparotomy was closed and the animal was recovered. The animals in Iteration I underwent intravenous subcutaneous port placement prior to recovery.

### Post-operative assessment

During follow-up, the pigs underwent weekly sedation procedures to obtain blood for analyses, such as complete blood counts (CBC), comprehensive metabolic panels (CMP), and echocardiographic (echo) assessment of the allograft. Tacrolimus trough levels were assessed using mass spectrometry (Duke University Hospital Pathology and Laboratory Services) every two days during the first two weeks after transplantation. The animals were examined daily for graft activity primarily by palpation and graded on a 0–4+ scale where 0 corresponded to no palpable contractility and 4+ corresponded to visible contractility on the skin surface (Supplementary Table S1). The pigs were survived until there was complete cessation of contractility of the donor heart as determined by a palpation grade of 0 that was confirmed using ultrasound.

The animals were euthanized after undergoing general anesthesia through induced cardiac arrest by delivery of del Nido cardioplegia (mixed at Duke Compounding Facility, Durham, NC) to the aortic root of the native heart (Supplementary Figure S2). Following arrest, both the native and transplanted hearts were excised and biopsies of each region were obtained [atria, ventricles, septa, subdivided by level (base, mid, apex)] and preserved by flash freezing, freezing in Tissue-Tek optimal cutting temperature (OCT) compound (Sakura, Torrance, CA), or fixation in 10% neutral buffered formalin (NBF). Biopsies of the left anterior descending artery (LAD), lungs, liver, spleen, and psoas muscle were also collected and preserved similarly.

### Transvenous endomyocardial biopsy

Endomyocardial biopsies were obtained on post-operative days (POD) 23 and 30. The biopsies were obtained transvenously as previously described (24). Briefly, the animals were sedated using general anesthesia and intubated for ventilation. A 5-Fr micropuncture sheath was used to catheterize the femoral vein and replaced with an 8-Fr sheath. A 7-Fr bioptome was then inserted into the sheath and guided into the right ventricle of the cardiac allograft using fluoroscopic and ultrasound guidance. Biopsies were then obtained and stored in NBF.

### Histology and immunohistochemistry

Paraffin embedded sections (5 µm thick) were stained with hematoxylin and eosin (H&E). All sections were assessed for AR by a cardiac pathologist using International Society of Heart and Lung Transplantation grading criteria (25). OCT embedded frozen 10 µm thick sections were stained for both CD3ɛ (Invitrogen MA1-90582, mouse, 1:150) to identify the presence of T cells, and CD8α (BioRad MCA6048GA, rabbit, 1:150) to identify the subset of cytotoxic T cells. Goat anti-mouse secondary polyclonal antibody conjugated to Alexa Fluor 594 (Abcam, Cat# ab150116, 1:300) and goat anti-rabbit secondary antibody conjugated to Alexa Fluor 488 (Abcam, Cat# ab150077, 1:300) were used for secondary staining. Imaging was done using a Zeiss 780 upright confocal microscope (Carl Zeiss Microscopy, White Plains, NY). PNC was detected using OCT embedded was detected using OCT embedded frozen sections and purified α-PNC monoclonal antibody clone 19D8 at 10 µg/ml for 1 h at room temperature (15, 26). An avidin-biotin amplification step and chromogenic detection (DAB) of α-mouse HRP-conjugated secondary antibody was used to visualize PNC localization and expression. Tissues were counter-stained with Mayer's hematoxylin and mounted with Cytoseal 60 (Thermo Fisher, Grand Island, NE) mounting media for imaging.

### Cardiac magnetic resonance imaging

CMR imaging studies were performed using a 3.0 T MAGNETOM Vida scanner (Siemens Healthineers, Erlangen, Germany) equipped with a 32-channel chest coil and 72-channel spine array as previously described (27). All animals were under general anesthesia with endotracheal intubation for mechanical ventilation throughout the duration of the procedures. Studies were performed prior to transplantation in both the donor and recipient, and on POD 19 for the recipient (Supplementary Video S1). Cine-CMR, T1 mapping, T2 mapping, and delayed enhancement CMR images were obtained. Velocity encoded images were acquired near the anastomotic sites for the donor heart to verify patent blood flow to the implanted heart.

### Immunophenotyping

Flow cytometry was performed on peripheral blood mononuclear cells (PBMC) isolated from whole blood samples using LeucoSep density centrifugation tubes (Greiner Bio-One, Monroe, NC). Frozen PBMCs were thawed and washed with complete RPMI buffer comprised of RPMI 1,640 medium (Thermo Fisher Scientific, Waltham, MA), penicillin-streptomycin (10,000 U/ml) (Thermo Fisher Scientific, Waltham, MA), and fetal bovine serum (FBS) (Sigma-Aldrich, St. Louis, MO), followed by Fluorescent Activated Cell Sorting and Immunofluorescence Staining Buffer with FBS (Rockland Immunochemicals, Pottstown, MA). The washed PBMCs were then incubated with antibodies and reagents to identify live porcine leukocytes (CD45^+^, “live”), which were further divided into subsets: helper T cells (CD3^+^CD4^+^), cytotoxic T cells (CD3^+^CD8^+^), regulatory T cells (CD3^+^CD4^+^CD25^+^), B cells (CD3^−^CD21^+^), and natural killer cells (CD3^−^CD21^−^CD56^+^) as outlined in Supplementary Table S2. A FACSCanto-II cytometer (BD Biosciences, Franklin Lakes, NJ) was used to analyze the stained PBMC samples. All data was analyzed using FlowJo 10.10.0 (BD Biosciences, Franklin Lakes, NJ). The cell identification strategy is outlined in Supplementary Table S3 and Figure S2.

### Genomic and cell-free DNA library preparation and sequencing

Whole genome sequencing was used to analyze blood samples for genomic and cfDNA. Genomic DNA (gDNA) was isolated from whole blood samples that were flash frozen and stored at −80°C. gDNA was isolated from these samples after thawing using the Promega ReliaPrep Blood gDNA Miniprep System (Madison, WI). For cfDNA isolation, plasma was isolated from whole blood samples following centrifugation at 850×g for 10 min. Samples were flash frozen prior to storage at −80°C. cfDNA was isolated from these samples after thawing using the QIAGEN QIAamp DSP Circulating NA Kit (Hilden, Germany). The purified gDNA and cfDNA samples were stored at −20°C prior to sequencing. The Duke Sequencing and Genomics Technologies Core Facility performed genome sequencing on both gDNA and cfDNA samples using the KAPA HyperPrep Kit (Cat# 07962363001, Roche, Basel, Switzerland) to build the DNA libraries and used the NovaSeq 6000 (Illumina, San Diego, CA) to perform the DNA sequencing. Reads were aligned to the Sscrofa11.1 version of the porcine genome with the BWA algorithm (28). Alignment processing and variant calling were performed using the GATK toolkit following the Broad Institute's Best Practices Workflow (29, 30).

### Statistical analysis

Statistical methods are detailed in the figure legends. Nonparametric data was assessed using Mann–Whitney U test when results were independent, or using Wilcoxon signed-rank test when data were pair-matched. These analyses were performed using GraphPad Prism version 10.3.1 (509) for Windows (GraphPad Software, Boston, MA). FlowJo 10.10.0 (BD Biosciences, Franklin Lakes, NJ) was used to generate spectral plots of flow cytometry data and uniform manifold approximation and projection (UMAP) plots to analyze these data across multiple animals and timepoints (31). Cell population analyses were performed by calculating super-enhanced Dmax subtraction (SE Dymax %) to calculate the percentage of positive cells found in the endpoint vs. the baseline timepoint. Chi-squared analysis was used to determine statistical differences between the samples. Statistical notations used in the figures: *p* > 0.05, not significant (ns); *p* < 0.05, *; *p* < 0.01, **; *p* < 0.001, ***; and *p* < 0.0001, ****.

## Results

### Degree of SLA mismatch and peri-operative immunosuppression affect time to fulminant acute rejection

Detailed results of the SLA genotyping are presented in Supplementary Table S4. A total of 15 heterotopic heart transplantations were performed, of these 3 pigs did not survive past 24 h due to complications from hemorrhagic shock after surgery. Two different degrees of SLA mismatch were assessed: partial mismatch (Iteration I) and complete mismatch (Iteration II and III). Iteration I comprised 2 pigs that had a total of 3 Class I antigenic mismatches and 1 Class II antigenic mismatch with their pairs. Iteration II comprised 2 pigs and Iteration III comprised 8 pigs (Animals A-H); all of the pairs had 4–5 Class I antigenic mismatches and 2–3 Class II antigenic mismatches. Iterations I, II, and III are summarized in Table 1. Each iteration differed in choice of calcineurin inhibitor (CNI), dosing of the CNI, duration of peri-operative immunosuppression drug administration, and route of CNI administration.

**Table 1:** Protocol iteration details.

Protocol iteration	Degree of SLA mismatching	Immunosuppression medications	Pre-op CNI	Immunosuppression duration	Route of administration of CNI
I	Partial haplotype	Cyclosporine Methylprednisolone MMF	No	21 days	IV
II	Complete haplotype	Tacrolimus Methylprednisolone MMF	Yes, 3 days	10 days	IM
III	Complete haplotype	Tacrolimus Methylprednisolone MMF	Yes, 3 days	14 days	IM

SLA, swine leukocyte antigen; IV, intravenous; IM, intramuscular; CNI, calcineurin inhibitor; MMF, mycophenolate mofetil.

Iteration I used cyclosporine administered intravenously (IV) through a subcutaneous venous port and dosed 10–20 mg/kg daily with a therapeutic trough blood level target of 100–300 ng/ml. Cyclosporine, methylprednisolone and mycophenolate mofetil (MMF) were administered for 21 days. A major challenge encountered with cyclosporine was that the trough levels were difficult to target within the therapeutic range. Therefore, for Iteration II and III, tacrolimus was administered intramuscularly (IM) and dosed 0.01–0.10 mg/kg daily with a therapeutic trough blood level target of 5–15 ng/ml. Tacrolimus, methylprednisolone and MMF were administered for 10-days (Iteration II) or 14-days (Iteration III) (Supplementary Figure S3). To validate the therapeutic range for tacrolimus, one pig from Iteration III was dosed subtherapeutically (Animal H) for a target trough blood level of 1–4 ng/ml, while another pig was dosed supratherapeutically (Animal F) for a target trough blood level 16–25 ng/ml. The serum trough levels were checked every other day while the animal was receiving either drug. Use of tacrolimus rather than cyclosporine proved to be more consistent and less complex with a more desirable dosing profile requiring only once daily dosing and consistent trough levels between doses.

Graft survival outcomes are summarized in Table 2. The pigs in Iteration I did not reach fulminant rejection or demonstrate clinical signs of allograft rejection at days 79 and 87 and were euthanized at those time points, respectively. The protocol utilized in Iteration II resulted in fulminant graft rejection at 17 and 23 days post-transplantation. To understand the influence of increased time of immunosuppression on graft survival, Iteration III was developed by increasing the time of immunosuppression from 10 to 14 days. Protocol Iteration III resulted in a median graft survival time of 41 days with an interquartile range of 39–53 days.

**Table 2:** Detailed summary of iteration design and outcomes.

Animal ID	Iteration	Blood type	Donor SLA haplotype	Recipient SLA haplotype	Mismatch	CNI	Immuno-suppression Days	Days to Endpoint^a^	Histology Grade at Endpoint	CAV
	I	A	4.5/7.8	4.5/4.5	Partial	Cyclosporine	21	Not reached (sac 87 days)	No rejection	
	I	A	4.5/7.8	4.5/4.5	Partial	Cyclosporine	21	Not reached (sac 79 days)	Moderate to Severe rejection	
	II	A	6.7/7.8	4.5/5.6	Complete	Tacrolimus	10	22	Severe rejection	
	II	Non-A	4.5/5.6	6.7/92.39	Complete	Tacrolimus	10	17	Severe rejection	
A	III	A	4.5/5.6	6.7/6.7	Complete	Tacrolimus	14	41	Severe rejection	Yes
B	III	Non-A	4.5/5.6	6.7/6.7	Complete	Tacrolimus	14	89	Severe rejection	Yes
C	III	Non-A	4.5/5.6	6.7/7.8	Complete	Tacrolimus	14	33	Severe rejection	Yes
D	III	Non-A	4.5/5.6	6.7/6.7	Complete	Tacrolimus	14	53	Severe rejection	Yes
E	III	A	4.5/5.6	6.7/6.7	Complete	Tacrolimus	14	39	Severe rejection	Yes
F	III	A	5.6/6.7	4.5/4.5	Complete	Tacrolimus (supratherapeutic)	14	Not reached (sac 106 days)	No global rejection; areas of mild to moderate rejection	No
G	III	A	5.6/7.8	4.5/6.7	Complete	Tacrolimus	14	42	Severe rejection	
H	III	A	4.5/5.6	6.7/92.39	Complete	Tacrolimus (subtherapeutic)	14	25	Severe rejection	

^a^ Endpoint consisted of cessation of graft contractility, confirmed by palpation and echocardiography.

In the context of Iteration III, when subtherapeutic tacrolimus dosing was used, a decreased survival time of 24 days was observed. In contrast, when supratherapeutic tacrolimus dosing was used, there was an extended survival time beyond 100 days. Of note, the pig dosed supratherapeutically demonstrated microinfarcts scattered throughout the myocardium on CMR and on final pathology, suggestive of a shower microembolic event soon after transplantation. The remainder of the results presented pertain to Iteration III outcomes.

### Changes in palpation grading, troponin measurements, echocardiography, and evidence of lymphocytic infiltration on histology demonstrate progression of acute rejection

**Figure 2:**
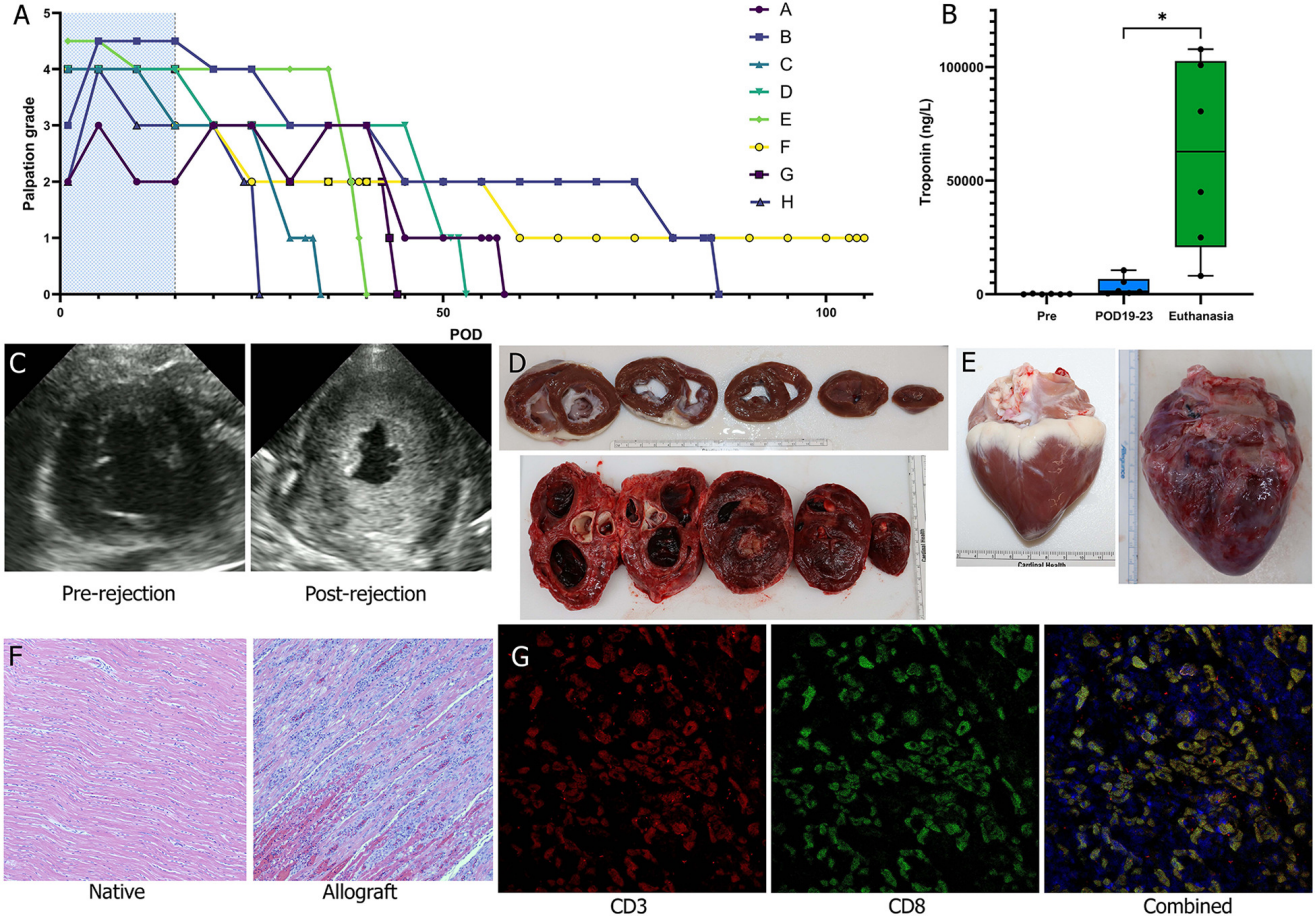
Histologic and functional changes of acute rejection. **(A)** Palpation grade was assessed 3–4 times daily for each animal during the observation period (peri-operative immunosuppression period shaded in blue). **(B)** Troponin levels were measured 2–3 times a week and were significantly greater at the time of fulminant acute rejection relative to POD 19-23 and baseline (Wilcoxon matched-pairs signed rank test). **(C)** Echocardiography was used as an adjunct to palpation grading to assess for cessation of cardiac activity and qualitative trending of LV wall thickness. **(D)** Cross sections were examined following explantation of the animal's native heart (top) and of the allograft (bottom). By the time of fulminant acute rejection there was pronounced LV hypertrophy. **(E)** There was also notable scarring and inflammation of the allograft (bottom) at time of explantation noted on gross pathology. **(F)** On H&E there was significant lymphocytic infiltration and myocardial degeneration; images shown were taken at 10× magnification. **(G)** Immunofluorescent staining of the allograft tissue for CD3 and CD8 identified cytotoxic T cell infiltration of tissues; images shown were taken at 20× magnification.

Traditional measures utilized to monitor AR were physical examination through palpation of the beating graft, measurement of troponin levels in the blood, echo to assess contractility and muscle morphology, and tissue biopsies collected transvenously, and at the time of euthanasia (Figure 2). Palpation grading was the principal screening method and was performed approximately every 8–12 h. Grading tended to vary between animals, depending on the final positioning of the heart in the abdomen. As a result, the beating graft was easier to palpate in some animals vs. others. However, there was no notable qualitative difference in contractility observable on echocardiography.

High sensitivity troponin I blood levels were evaluated throughout the survival period of each animal. A significant difference was observed between troponin level measurements from POD19-23 [median (Q1, Q3): 1,301 (586, 6,728) ng/L] vs. those collected within 5 days of euthanasia [62,747 (20,779, 102,573) ng/L] indicating significant cardiac injury attributable to fulminant allograft rejection (*p* = 0.03, *n* = 6). Animal F that was dosed with supratherapeutic levels of tacrolimus did not demonstrate an increase in troponin, with a baseline level of 546 ng/L but a level of 111 ng/L at the time of euthanasia. Conversely, for animal H that was dosed subtherapeutic tacrolimus, there was a large increase in troponin at the time of euthanasia, consistent with cardiac injury from AR.

### T cell mediated acute rejection with CD8 predominance on myocardial histology at euthanasia

H&E histological analysis demonstrated severe grade AR (ISHLT 3R) in 7 of the 8 grafts at the time of euthanasia (Table 3). Only the graft from the pig that received supratherapeutic tacrolimus dosing (Animal F) demonstrated no rejection, with only focal areas of mild to moderate inflammation. There was no evidence of rejection in any of the respective native hearts. Endomyocardial biopsies at POD 23 were collected from animals A-F (*n* = 6) and at POD 30 from animals C-G (*n* = 5). Of the biopsies collected at POD 23, 2 were non-diagnostic (A and B) because they lacked myocardial tissue; 2 demonstrated severe grade rejection (ISHLT 3R) (D and E); and 2 demonstrated no rejection (ISHLT 0R) (C and F). One biopsy collected at POD 30 was non-diagnostic (G). Animal F again demonstrated no rejection at POD 30 (ISHLT 0R), and animals C-E demonstrated moderate or severe grade rejection (ISHLT 2R-3R) at POD 30. Immunofluorescent staining for T cells (CD3^+^) and cytotoxic T cells (CD3^+^CD8^+^) was performed on tissue biopsies collected at the time of euthanasia for animals A-E. T cells and cytotoxic T cell subsets were visualized in all graft tissues but were absent in all corresponding native heart tissues (Figure 2G).

**Table 3:** Myocardial histological analysis results.

Animal	ISHLT grade POD 23	ISHLT grade POD 30	ISHLT grade euthanasia native heart	ISHLT grade euthanasia graft	T cells (CD3/CD8) euthanasia
A	Non-diagnostic	n/a	No Rejection (0R)	Acute Rejection (3R)	+
B	Non-diagnostic	n/a	No Rejection (0R)	Acute Rejection (3R)	+
C	No Rejection (0R)	Acute Rejection (2R)	No Rejection (0R)	Acute Rejection (2R-3R)	+
D	Acute Rejection (3R)	Acute Rejection (2R)	No Rejection (0R)	Acute Rejection (3R)	+
E	Acute Rejection (3R)	Acute Rejection (3R)	No Rejection (0R)	Acute Rejection (3R)	+
F	No Rejection (0R)	No Rejection (0R)	No Rejection (0R)	No Rejection; focal areas of inflammation	n/a
G	n/a	Non-diagnostic	No Rejection (0R)	Acute Rejection (3R)	n/a
H	n/a	n/a	No Rejection (0R)	Acute Rejection (2R-3R)	n/a

n/a, not available.

**Table 4:** Post-transplantation CMR results.

Measurement	Native (*n* = 5)	Allograft (*n* = 5)	*p*-value
ΔLV mass, g (mean ± SD)	8.3 ± 2.5	63.5 ± 16.8	<0.0001
ΔT1 mapping, ms	77.8 ± 41.9	222.6 ± 55.0	0.002
ΔT2 mapping, ms	6.0 ± 3.1	13.7 ± 4.2	0.008
Delayed enhancement, %	0	6.3 ± 6.2	0.008

Animal/allograft that did not progress to fulminant rejection excluded from analyses.

### CMR provides high sensitivity for detecting progression of acute rejection during survival

**Figure 3:**
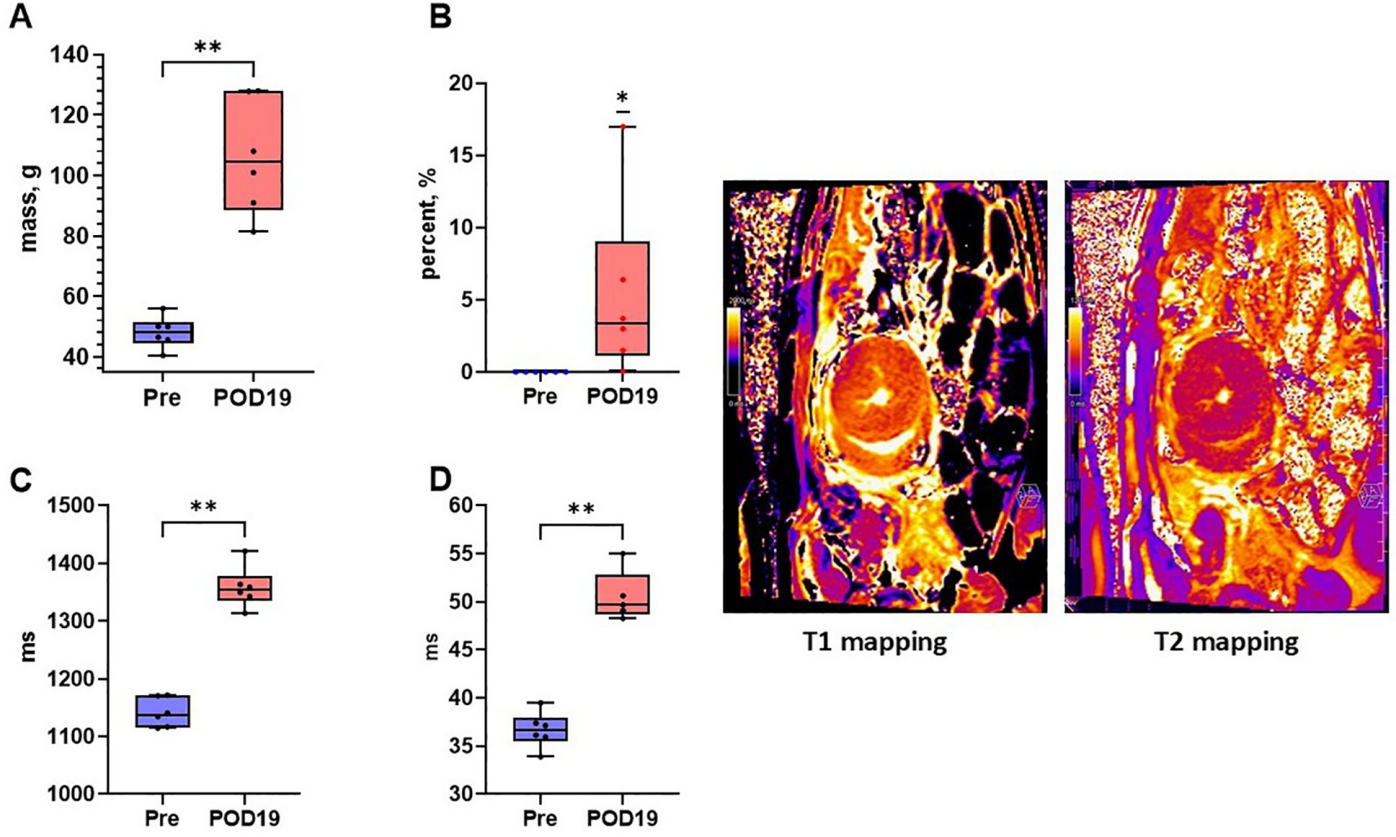
Cardiac magnetic resonance imaging detects early changes of acute rejection. **(A)** LV mass (Mann–Whitney U test), **(B)** late gadolinium enhancement (Wilcoxon signed rank test), **(C)** T1 mapping (Mann–Whitney U test), and **(D)** T2 mapping (Mann–Whitney U test) were all significantly increased by post-operative day 19 relative to baseline indicating tissue changes attributable to acute rejection. Representative T1 mapping and T2 mapping cross sections of a heterotopically transplanted heart are demonstrated.

CMR analysis demonstrated a significant increase in several measures correlated with progression of AR (Figure 3 and Table 4). LV mass (grams, g) was significantly increased by POD 19 [median (Q1, Q3): Pre, 48.25 (45.83, 50.08)g; POD 19, 104.5 (93.5, 122.93)g, *n* = 6, *p* = 0.002]. LV mass in Animal F increased as well from 63.1 g to 109.3 g. T1 mapping (milliseconds, ms) was significantly increased [median (Q1, Q3): Pre, 1,137.55 (1,120.9, 1,163.19)ms; POD 19, 1,354.15 (1,344.3, 1,362.35)ms, *n* = 6, *p* = 0.002]. T1 mapping also increased in Animal F, however, also to a lesser extent from 1,184.24 ms to 1,285.84 ms which was similar to the increase seen in the animal's native heart (1,128.86 ms to 1,194.40 ms). T2 mapping increased by POD 19 [median (Q1, Q3): Pre, 36.65 (36.00, 37.32)ms; POD 19, 49.68 (49.1, 50.64)ms, *n* = 6, *p* = 0.004] in all the animals. There was a similar increase in T2 mapping observed in Animal F from 36.76 ms to 46.66 ms. Lastly, delayed enhancement following gadolinium administration indicative of fibrosis was also significantly greater by POD 19 [median (Q1, Q3): Pre, 0.00%; POD 19, 3.35% (1.88%, 5.73%), *n* = 6, *p* = 0.03]. Delayed enhancement was also increased in Animal F (0.00–9.7%), however, the pattern of scar distribution was more consistent with a shower microembolic event rather than rejection.

### Circulating donor-derived cell free DNA is detectable and elevated and peripheral blood mononuclear cell immunophenotyping demonstrates elevated CD8 T cell levels during acute rejection

**Figure 4:**
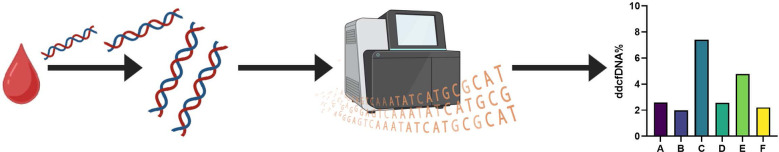
Donor derived cell-free DNA percentage is detectable at fulminant graft rejection. Cell free DNA was isolated from blood samples collected from recipient animals. The isolated DNA was then sequenced and analyzed to assess for detectable levels of ddcfDNA. Comparable levels of ddcfDNA were detectable in all of the evaluated animals, including the animal that did not progress to fulminant graft rejection within the designated study timeframe.

**Figure 5:**
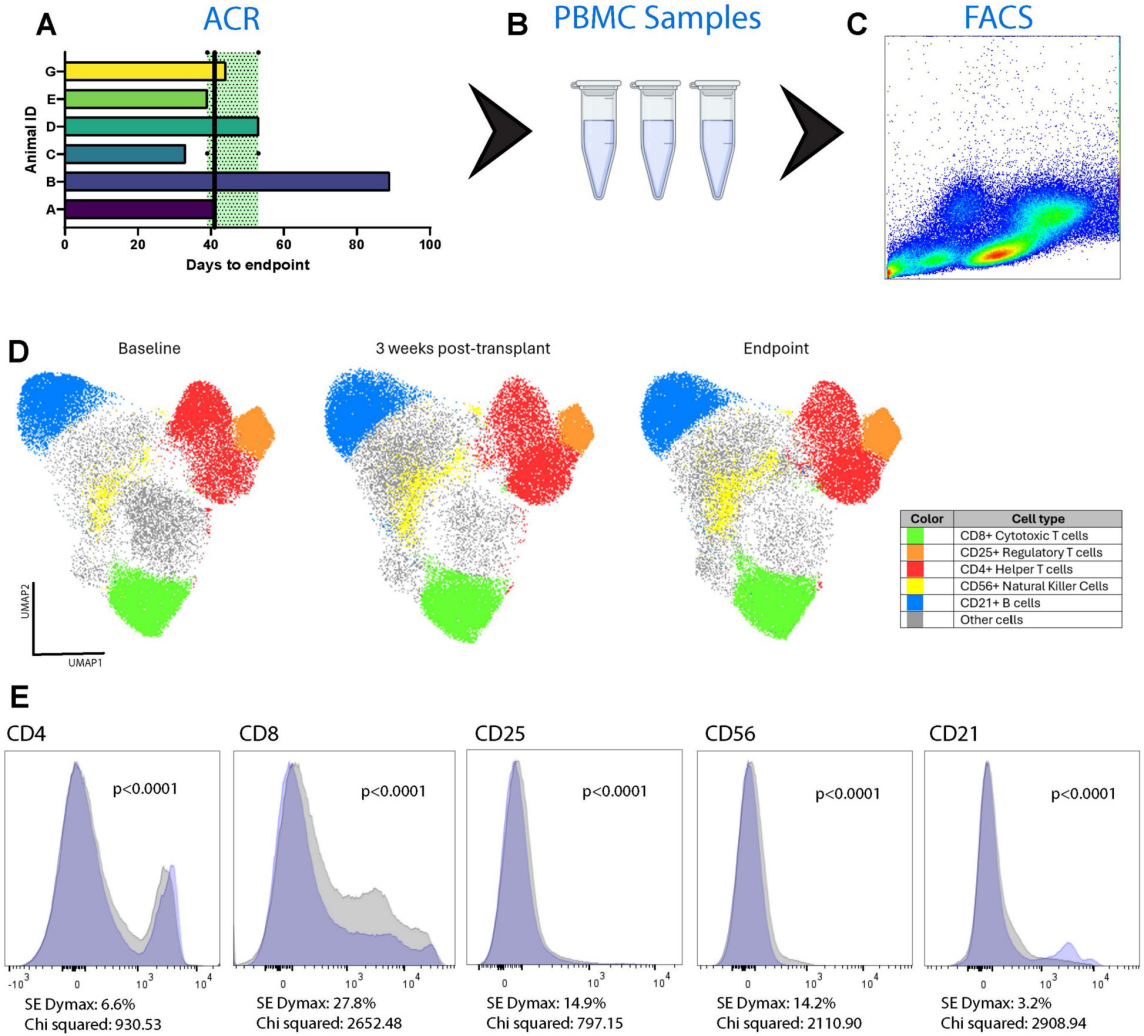
Expansion of T cells in the recipient animals’ circulation over time*.* Blood samples were collected over the course of each animal's observation period **(A)** Peripheral blood mononuclear cells (PBMC) were isolated **(B)** and used for immunophenotyping through flow cytometry **(C)** Overall cell population changes were pooled from Animals A–E and G using uniform manifold approximation and projection (UMAP) analysis **(D)** Three timepoints were assessed: baseline, 3 weeks post-transplantation, and at the time of cessation of graft activity. Relative to baseline, at the endpoint there was significant expansion of CD4^+^ (6.6%) and CD8^+^ (27.8%) T cells. There was also significant expansion of CD25^+^ T cells (14.9%), CD56^+^ natural killer cells (14.2%), and CD21^+^ B cells (3.2%). The changes in specific cell subtypes from baseline to endpoint are also shown as plots with the blue area corresponding to the baseline and the grey area corresponding to the endpoint **(E)**.

Donor-derived cfDNA (ddcfDNA) analysis was performed on 6 animals (Animals A-F). ddcfDNA levels were measurable in all animals with a median of 2.59% and interquartile range of 2.55%–4.77% (Q1–Q3) in the animals who developed fulminant rejection (Animals A-E) (Figure 4). Animal F had a comparable ddcfDNA level of 2.21%, despite not reaching the endpoint of fulminant rejection, potentially indicating that there was subclinical rejection developing in the allograft. Immunophenotyping of circulating PBMCs in Animals A-E demonstrated significant increases in CD4^+^, CD8^+^, CD25^+^, CD56^+^, and CD21^+^ at the time of fulminant ACR (*p* < 0.0001) (Figure 5). Of these, the greatest increase was in CD8^+^ T cells by 27.8% (Chi-squared = 2,652.48). This increase was also observed in Animal F by 43.0% (Chi-squared = 4,900.47, *p* < 0.0001) despite not demonstrating histologic or functional changes of AR.

### Pro-N-Cadherin as a novel marker for rejection-associated tissue injury

**Figure 6:**
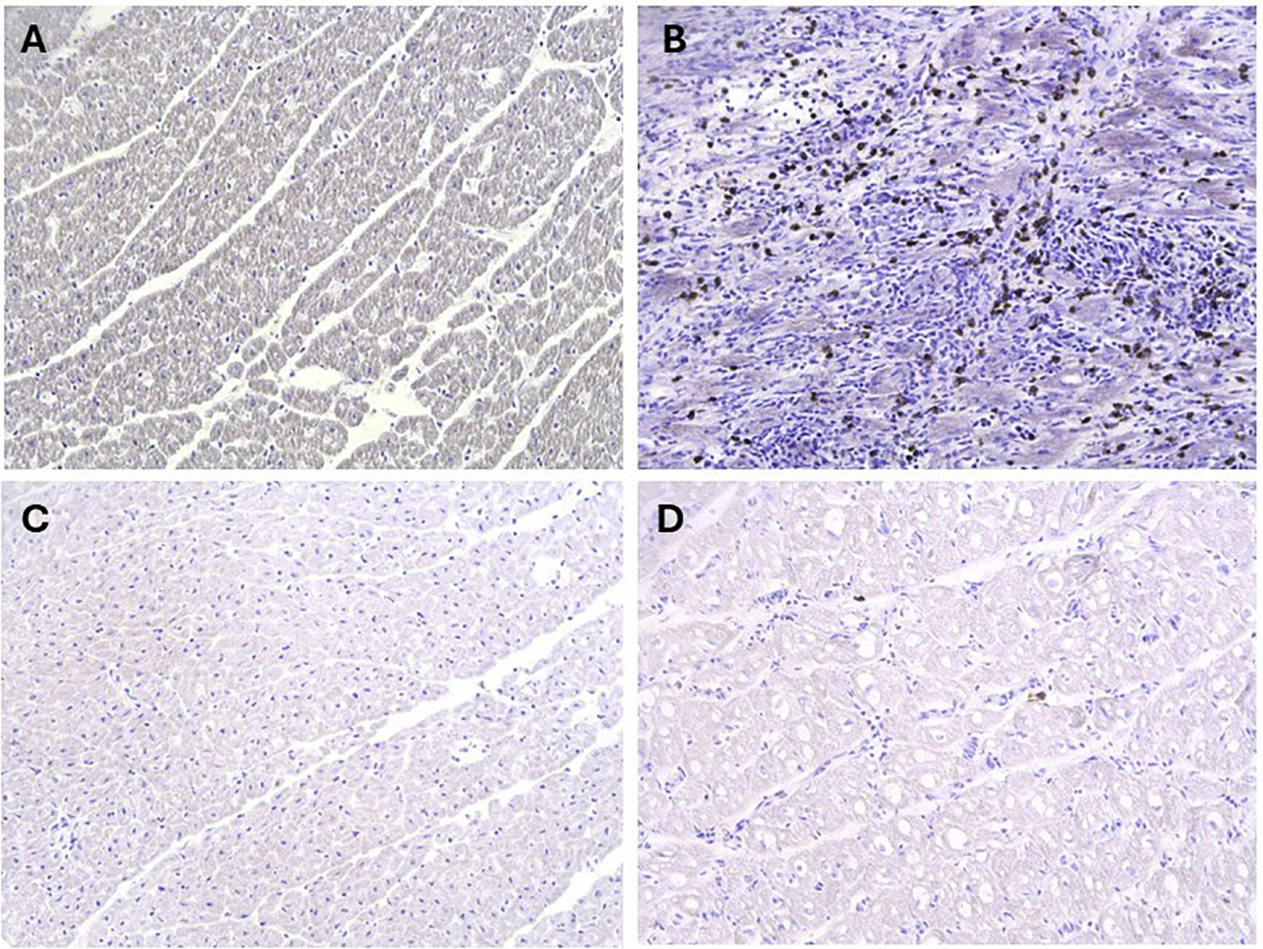
Assessment of Pro-N-cadherin as a marker of acute rejection. Immunohistochemistry analysis for PNC had no evidence of the marker on myocardial biopsies of the native heart **(A)**, however had abundant staining for PNC on myocardial biopsies of the transplanted hearts that progressed to fulminant acute rejection **(B)** In comparison, there was negligible to no evidence of PNC on the both the native heart **(C)** and transplanted heart **(D)** myocardial biopsies from the animal that did not progress to fulminant acute rejection (Animal F).

The presence of PNC was assessed for in the context of rejection-associated tissue injury using animals A-F from Iteration III. PNC was observed in rejected allograft heart tissues (Animals A-E), and absent in native heart tissues (Figures 6A,B). When assessed in the non-rejected allograft from Animal F, PNC was not observed in either the allograft or the native heart (Figures 6C,D).

## Discussion

Systematic, reproducible models of disease are needed for the development of effective and safe therapies (32). We present a detailed characterization of a clinically relevant porcine model of ACR. Our group previously demonstrated that normothermic *ex vivo* machine perfusion through use of the TransMedics OCS can serve as a platform for administering viral vectors to porcine hearts to achieve effective gene delivery throughout the entire organ, enabling a future for therapeutic applications (11, 22). For that reason, we chose to incorporate the OCS into this model characterization vs. utilizing traditional static cold storage methods. Notwithstanding, future expansion of this model characterization could be done to examine characteristics of AR when traditional static cold storage is utilized instead of *ex vivo* machine perfusion.

We examined the influence that different degrees of SLA mismatching had on timing to fulminant AR post-transplantation, such that Iteration I utilized partially mismatched pairs vs. Iterations II and III which utilized fully mismatched pairs. Selecting for partial SLA mismatching in conjunction with a longer (21 day) post-transplantation immunosuppression period in Iteration I resulted in a longer survival period for the graft. However, selection for full SLA mismatching in conjunction with a shorter (10 day) post-transplantation immunosuppression period in Iteration II resulted in a shorter survival period for the graft. We examined the flexibility of this model to accommodate a longer time to fulminant graft rejection in Iteration III by administering a longer immunosuppression period. Iteration III resulted in a longer time to fulminant graft rejection. This mirrors what was described by Madsen et al., who also described a similar model of AR. Complete SLA mismatch led to severe AR with survival time between 7 and 8 days, however with the addition of 11 days of therapeutic cyclosporine post-transplantation the allograft survival time extended to 20–23 days (5).

The immunosuppression regimen of this study was selected to closely mirror what is commonly used in clinical practice. While immunosuppression protocols can vary between institutions, they generally comprise of a CNI, like tacrolimus; an antimetabolite, like mycophenolate mofetil; and a tapering dose of a glucocorticoid over the first year after transplantation. The main difference is that in clinical practice, systemic immunosuppression drugs are continued long-term rather than terminated after a few weeks following transplantation. One of the challenges encountered during the design of this model was determining the ideal CNI regimen. Both cyclosporine and tacrolimus have been described to induce prolonged allograft survival in pigs following organ transplantation, particularly when administered at higher doses (33–36). Additionally, pigs metabolize and eliminate tacrolimus from their circulation at different rates than humans (37). To validate the therapeutic dosing range of tacrolimus, we examined what would happen to the rate of fulminant graft rejection when tacrolimus was dosed subtherapeutically or supratherapeutically in Iteration III. We found that when tacrolimus is dosed to a target range below 5 ng/ml, progression to fulminant graft rejection was accelerated. However, when tacrolimus was dosed to a target range greater than 15 ng/ml we observed a substantially prolonged allograft survival time following transplantation. However, since these observations derived from one pig each, further experiments to validate these findings are required.

A biologic hallmark of ACR is the expansion of CD4^+^ and CD8^+^ T cell subsets that drive adaptive immune responses (38, 39). While the porcine immune system has been characterized as closely resembling that of the human immune system, detailed immunological differences that drive AR are yet to be well described (7). We characterized the expansion of these T cell subsets in Iteration III through flow cytometry and immunofluorescent tissue staining. There were increases in both CD4^+^ and CD8^+^ levels with the more notable increase in CD8^+^ levels. In conjunction, we observed severe grade rejection on all histologic tissue sections collected from the animals that progressed to cessation of graft contractility. A possible additional measurement to incorporate into this model moving forward could be quantitative analysis of T cell infiltration (cells/mm^2^) to help evaluate inter-animal variations in greater definition. Notwithstanding, additional analyses are required to further understand the immunological nuances that might affect the direct translatability of this preclinical porcine model. We also applied advanced methods for characterizing AR in this model, including CMR and ddcfDNA quantification. While CMR proved to be sensitive for detecting these changes, we did not observe the same sensitivity when quantifying ddcfDNA in the recipient circulation. Similar levels of ddcfDNA were detectable in all of the animals in Iteration III, including Animal F.

This report is the first to assess and observe PNC-positive cell infiltration in tissue that has undergone AR. While the identity of the PNC-expressing cells in this model remains unclear, prior studies have shown aberrant cell surface expression of PNC on activated fibroblasts implicating it in cellular migration processes (15). Activated fibroblasts are the major contributors of collagen deposition in tissue fibrosis. AR often leads to significant tissue fibrosis, which was also observed on CMR and on histological analyses in this report. Further studies are needed to validate the identity of the PNC-positive cells observed in this model and elucidate the biological function of PNC in AR.

We observed several interesting factors in the pig that did not experience progression to cessation of graft contractility within the allotted study timeframe. Despite not having evidence of severe AR on H&E assessment nor on physical exam, Animal F had comparable levels of ddcfDNA and of CD8^+^ detected in its circulation to the animals that did progress to fulminant rejection. It is plausible that Animal F was experiencing subclinical AR despite not yet experiencing major tissue injury. On review of haplotyping and genotyping data for each animal, there were no significant differences between Animal F and its donor relative to the other recipient and donor pairs that could account for its prolonged allograft survival. Additionally, the number of antigenic mismatches between Animal F and its donor matched the number of mismatches observed in the other pairs. For these reasons, we attribute Animal F's prolonged graft survival to receiving a supratherapeutic dosing of tacrolimus during the peri-operative immunosuppression period.

There is high utility for a porcine model of AR at this point in time as new therapeutics and preservations strategies are considered. The growing use of *ex vivo* machine perfusion in transplantation has expanded the number of organs being transplanted due to the ability to recondition organs and extend the amount of time an organ can safely spend outside of a body. In parallel, advanced therapies have been progressively expanding into the clinical realm allowing for unprecedented achievements in medicine, such as the first genetically modified porcine xenotransplant into a human recipient. Applications of gene editing and gene therapy are at the forefront of creating these bioengineered organs, supported in part by the advancement of *ex vivo* machine perfusion. This model will allow for robust and clinically relevant measures of AR that can be used in the development of therapeutics that can be safely translated into clinical interventions.

## Data Availability

The raw data supporting the conclusions of this article will be made available by the authors, without undue reservation.

## Supplementary material

Supplementary Figure S1 Representative image of native heart and intra-abdominal heart at time of endpoint.

Supplementary Figure 2 Gating strategy for immunophenotyping acute cellular rejection. All PBMC samples were initially processed by creating a spectral spread to exclude all debris from the sample **(A)**. Singlet cells were then isolated from this gate and another spectral spread was created **(B)**. Next, lymphocytes were resolved using CD45 **(C)** and were further resolved to only include live cells using a commercial LIVE-DEAD stain **(D)**. From this, T cells were resolved from B cells using CD3 and CD21, respectively **(E)**. T cells subsets were further resolved using CD4 to identify helper T cells and CD8 for cytotoxic T cells **(F)**. Using the CD4 subpopulation, CD25 was used to resolve the regulatory T cell population **(G)**. Lastly, the CD3-CD21- cells were gated in order to identify natural killer cells using CD56 **(H)**.

Supplementary Figure S3 Post-operative immunosuppression drug schedule for Iteration III. Methylprednisolone was administered as a taper starting at 24 mg/kg twice a day and ending at 0.14 mg/kg once daily either intramuscularly or orally. Mycophenolate mofetil was administered as 500 mg twice a day orally. Tacrolimus was administered to achieve a trough blood level between 5 and 15 ng/ml as a once daily intramuscular injection. Abbreviations: IM – intramuscular; PO – per os; BID – twice a day.

Supplementary Video S1 Intra-abdominal heterotopically transplanted heart in a Yucatan pig on cardiac magnetic resonance imaging 19 days after transplantation.

Supplementary Table S4 Detailed SLA genotyping analysis.
