# Supplementary figures and images for: A porcine model of acute rejection for cardiac transplantation
Source: Front Cardiovasc Med. 2025 Jul 18;12:1549377. doi: 10.3389/fcvm.2025.1549377 (PMC12313652; doi:10.3389/fcvm.2025.1549377)

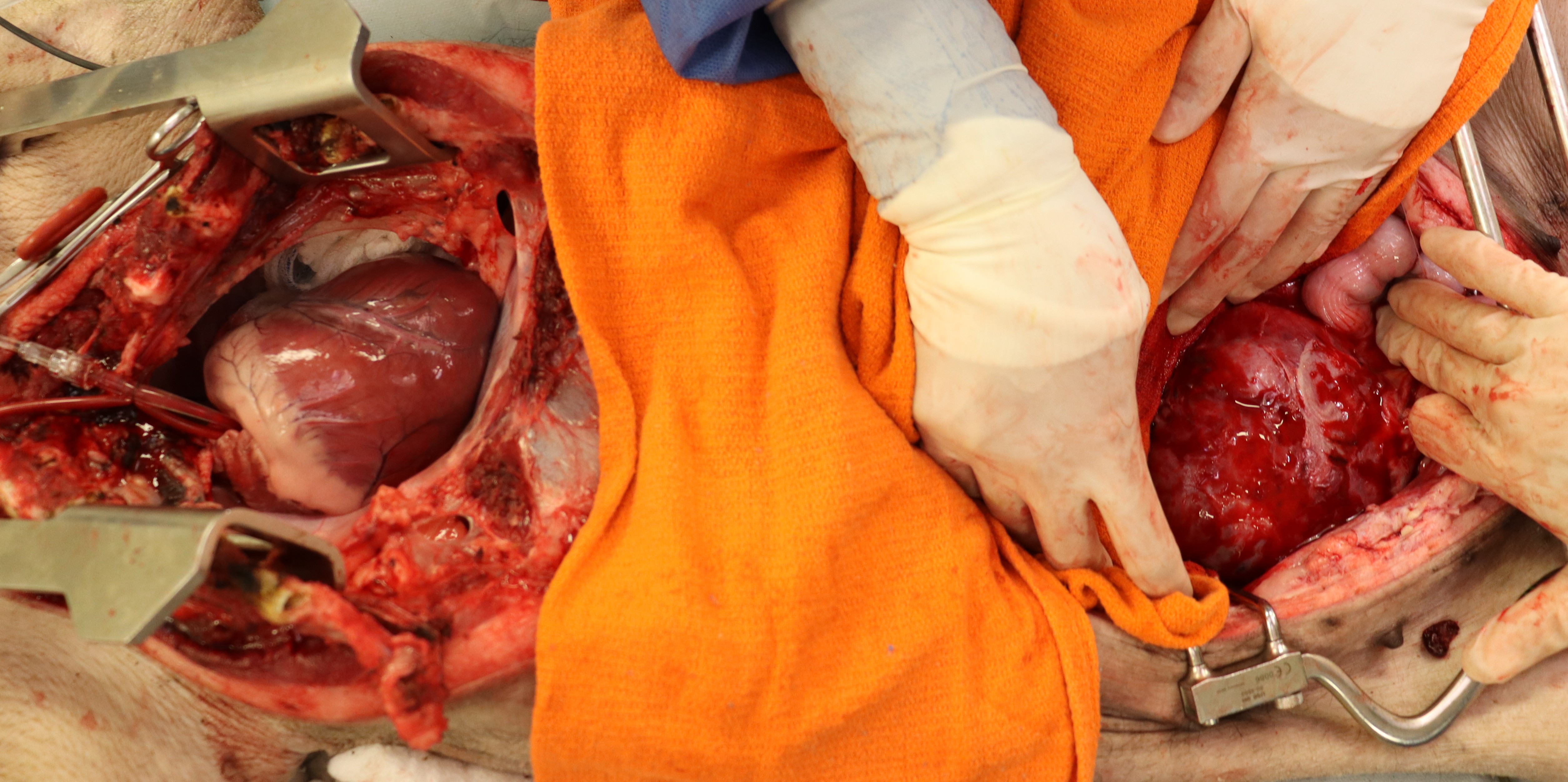

Supplement: Supplementary Figure S1 — Representative image of native heart and intra-abdominal heart at time of endpoint. [file Image1.jpg]

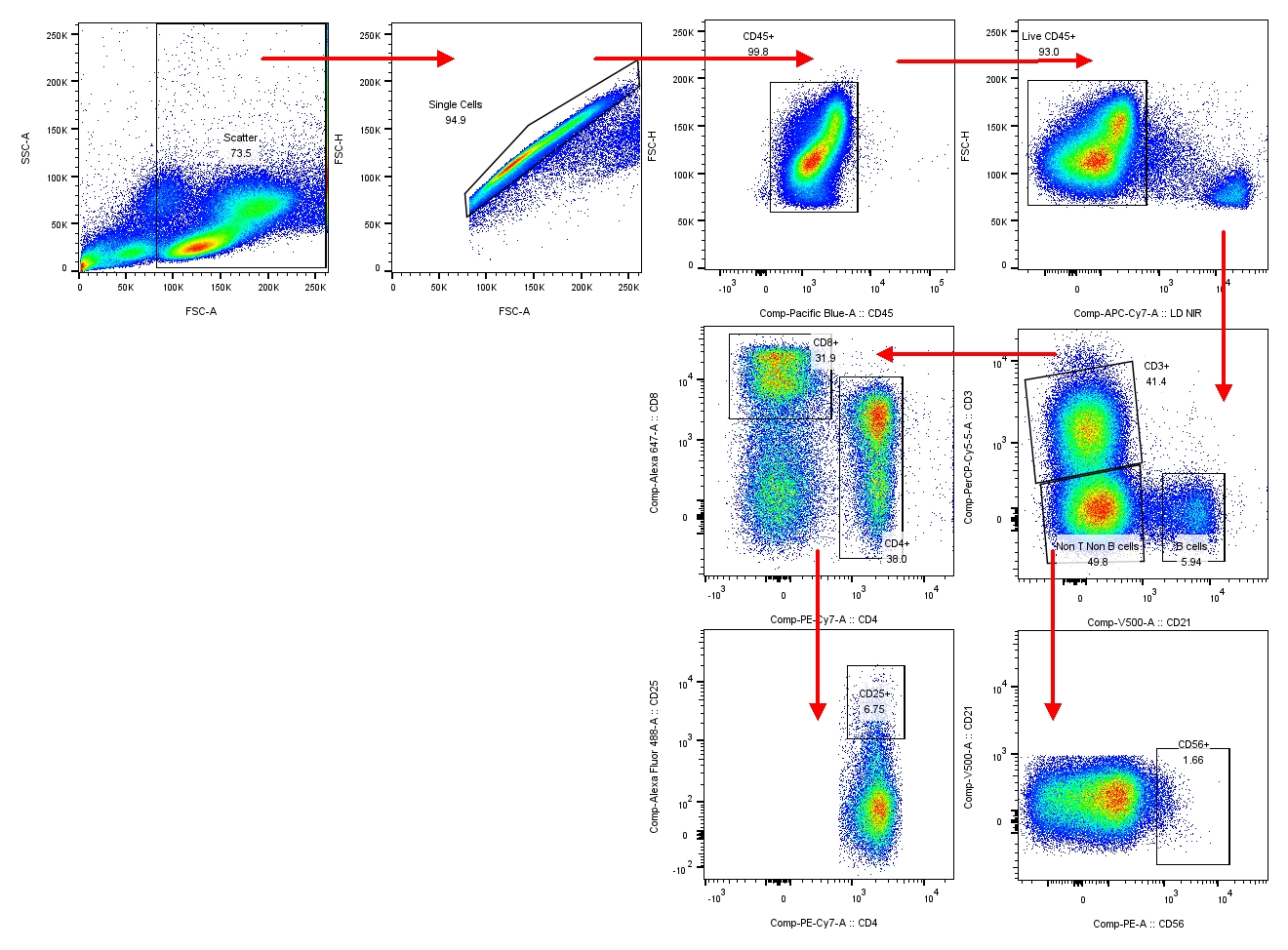

Supplement: Supplementary Figure 2 — Gating strategy for immunophenotyping acute cellular rejection. All PBMC samples were initially processed by creating a spectral spread to exclude all debris from the sample (A). Singlet cells were then isolated from this gate and another spectral spread was created (B). Next, lymphocytes were resolved using CD45 (C) and were further resolved to only include live cells using a commercial LIVE-DEAD stain (D). From this, T cells were resolved from B cells using CD3 and CD21, respectively (E). T cells subsets were further resolved using CD4 to identify helper T cells and CD8 for cytotoxic T cells (F). Using the CD4 subpopulation, CD25 was used to resolve the regulatory T cell population (G). Lastly, the CD3-CD21- cells were gated in order to identify natural killer cells using CD56 (H). [file Image2.jpg]

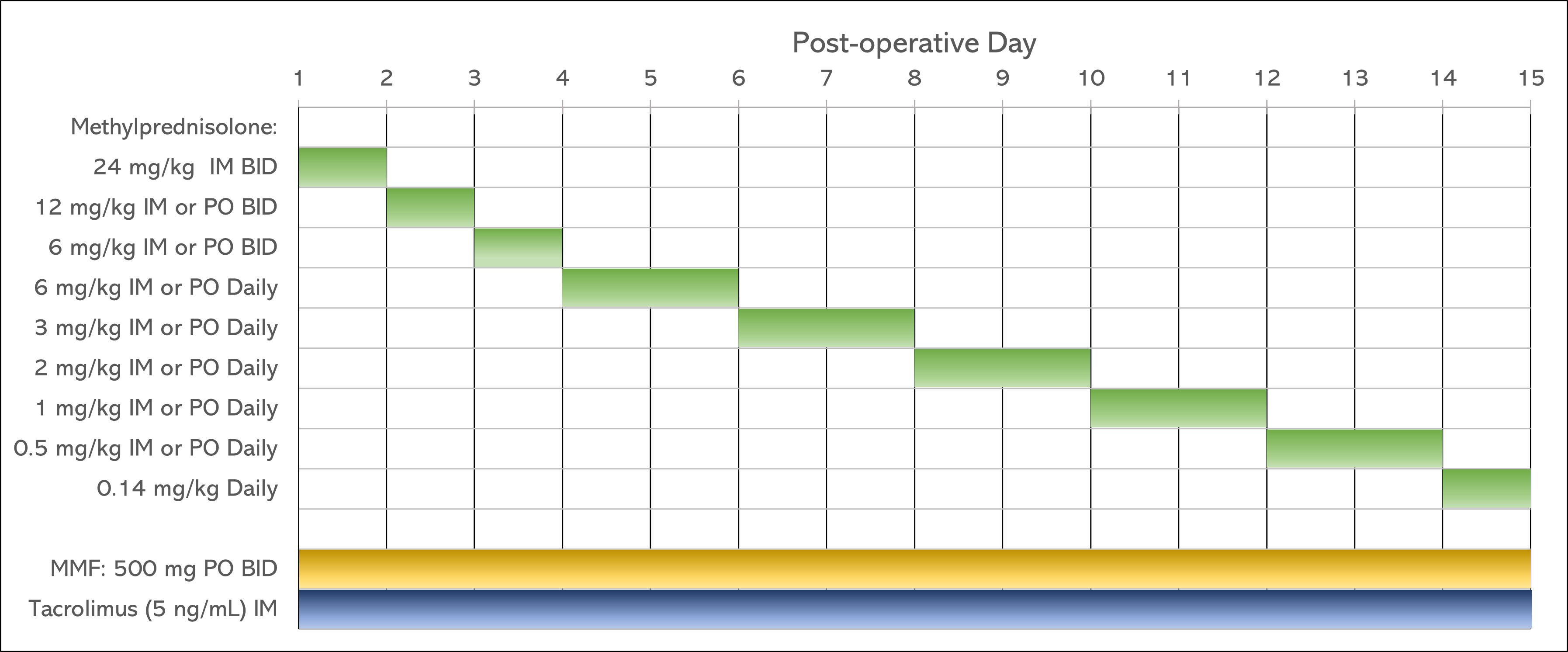

Supplement: Supplementary Figure S3 — Post-operative immunosuppression drug schedule for Iteration III. Methylprednisolone was administered as a taper starting at 24 mg/kg twice a day and ending at 0.14 mg/kg once daily either intramuscularly or orally. Mycophenolate mofetil was administered as 500 mg twice a day orally. Tacrolimus was administered to achieve a trough blood level between 5 and 15 ng/ml as a once daily intramuscular injection. Abbreviations: IM – intramuscular; PO – per os; BID – twice a day. [file Image3.jpeg]
