# Supplementary material for: A porcine model of acute rejection for cardiac transplantation
Source: Front Cardiovasc Med. 2025 Jul 18;12:1549377. doi: 10.3389/fcvm.2025.1549377 (PMC12313652; doi:10.3389/fcvm.2025.1549377)
Supplement: Supplementary Table S4 — Detailed SLA genotyping analysis. [file Table4.pdf]

	Animal ID	Study ID	Blood type	Haplotype	SLA-1	SLA-3	SLA-2	DRB1	DQB1	DQA	# of CI Ag MM (SLA-1/2/3)	# of CII Ag MM (DRB1/DQB1)
Donor	1081		A	4.5/7.8	04:01:01	04:01	04:02:01	05:01	02:01	02:02:02	**3**	**1**
					**08:01**	**07:01:01**	**05:02**	**08:01**	**02:02**	**02:03**		
Recipient	1018		A	4.5/4.5	04:01:01	04:01	04:02:01	05:01	02:01	02:02:02		
					04:01:01	04:01	04:02:01	05:01	02:01	02:02:02		
Donor	1110		A	4.5/7.8	04:01:01	04:01	04:02:01	05:01	02:01	02:02:02	**3**	**1**
					**08:01**	**07:01:01**	**05:02**	**08:01**	**02:02**	**02:03**		
Recipient	997		A	4.5/4.5	04:01:01	04:01	04:02:01	05:01	02:01	02:02:02		
					04:01:01	04:01	04:02:01	05:01	02:01	02:02:02		
Donor	2637		A	6.7/7.8	**08:05**	**06:01**	**05:04**	**06:01**	**06:01**	**01:06**	**4**	**3**
					**08:01**	**07:01:01**	**05:02**	**08:01**	**02:02**	**02:03**		
Recipient	2555		A	4.5/5.6	04:01:01	05:05	08:02	05:01	08:01	01:03		
					04:01:01	04:01	04:02:01	05:01	02:01	02:02:02		
Donor	2565		Non-A	4.5/5.6	**04:01:01**	**05:05**	**08:02**	**05:01**	**08:01**	**01:03**	**5**	**3**
					**04:01:01**	**04:01**	**04:02:01**	**05:01**	**02:01**	**02:02:02**		
Recipient	2557		A	6.7/92.39	08:05	06:01	05:04	06:01	06:01	01:06		
					19:03	03:12	20:01	07:XX	10:01	04:XX[2]		
Donor	3649	A	A	4.5/5.6	**04:01:01**	**05:05**	**08:02**	**05:01**	**08:01**	**01:03**	**5**	**3**
					**04:01:01**	**04:01**	**04:02:01**	**05:01**	**02:01**	**02:02:02**		
Recipient	3590	A	A	6.7/6.7	08:05	06:01	05:04	06:01	06:01	01:06		
					08:05	06:01	05:04	06:01	06:01	01:06		
Donor	4174	B	Non-A	4.5/5.6	**04:01:01**	**05:05**	**08:02**	**05:01**	**08:01**	**01:03**	**5**	**3**
					**04:01:01**	**04:01**	**04:02:01**	**05:01**	**02:01**	**02:02:02**		
Recipient	4188	B	Non-A	6.7/6.7	08:05	06:01	05:04	06:01	06:01	01:06		
					08:05	06:01	05:04	06:01	06:01	01:06		
Donor	4168	C	Non-A	4.5/5.6	**04:01:01**	**05:05**	**08:02**	**05:01**	**08:01**	**01:03**	**5**	**2**
					**04:01:01**	**04:01**	**04:02:01**	**05:01**	**02:01**	**02:02:02**		
Recipient	4204	C	Non-A	6.7/7.8	08:05	06:01	05:04	06:01	06:01	01:06		
					08:01	07:01:01	05:02	08:01	02:02	02:03		
Donor	4509	D	Non-A	4.5/5.6	**04:01:01**	**05:05**	**08:02**	**05:01**	**08:01**	**01:03**	**5**	**3**
					**04:01:01**	**04:01**	**04:02:01**	**05:01**	**02:01**	**02:02:02**		
Recipient	4482	D	A	6.7/6.7	08:05	06:01	05:04	06:01	06:01	01:06		
					08:05	06:01	05:04	06:01	06:01	01:06		
Donor	4492	E	A	4.5/5.6	**04:01:01**	**05:05**	**08:02**	**05:01**	**08:01**	**01:03**	**5**	**3**
					**04:01:01**	**04:01**	**04:02:01**	**05:01**	**02:01**	**02:02:02**		
Recipient	4517	E	A	6.7/6.7	08:05	06:01	05:04	06:01	06:01	01:06		
					08:05	06:01	05:04	06:01	06:01	01:06		
Donor	92-095	F	A	5.6/6.7	04:01:01	**05:05**	**08:02**	05:01	**08:01**	**01:03**	**5**	**3**
					**08:05**	**06:01**	**05:04**	**06:01**	**06:01**	**01:06**		
Recipient	92-103	F	A	4.5/4.5	04:01:01	04:01	04:02:01	05:01	02:01	02:02:02		
					04:01:01	04:01	04:02:01	05:01	02:01	02:02:02		
Donor	4906	G	A	5.6/7.8	04:01:01	**05:05**	**08:02**	05:01	**08:01**	**01:03**	**3**	**2**
					**08:01**	**07:01:01**	**05:02**	**08:01**	**02:02**	**02:03**		
Recipient	4878	G	A	4.5/6.7	04:01:01	04:01	04:02:01	05:01	02:01	02:02:02		
					08:05	06:01	05:04	06:01	06:01	01:06		
Donor	35232	H	A	4.5/5.6	**04:01:01**	**05:05**	**08:02**	**05:01**	**08:01**	**01:03**	**5**	**3**
					**04:01:01**	**04:01**	**04:02:01**	**05:01**	**02:01**	**02:02:02**		
Recipient	35225	H	A	6.7/92.39	08:05	06:01	05:04	06:01	06:01	01:06		
					19:03	03:12	20:01	07:XX	10:01	04:XX[2]		

Note:

Ag MM: Antigen Mismatch

[1] Allelic mismatch (highlighted in blue) is not considered as antigen mismatch (highlighted in red)

[2] High-resolution allelic typing not known
