# Supplementary material for: A porcine model of acute rejection for cardiac transplantation
Source: Front Cardiovasc Med. 2025 Jul 18;12:1549377. doi: 10.3389/fcvm.2025.1549377 (PMC12313652; doi:10.3389/fcvm.2025.1549377)
Supplement: Supplementary file 6 [file Table1.docx]

Supplemental Table 1: Palpation grading scale

| **Grade** | **Description** |
| --- | --- |
| 0 | No palpable contractility |
| 1 | Faint contractility, not easily palpable |
| 2 | Weak contractility, easily palpable |
| 3 | Moderate contractility palpable |
| 4 | Strong contractility palpable |
| 4+ | Visible contractility at the skin level |
