# Supplementary material for: A porcine model of acute rejection for cardiac transplantation
Source: Front Cardiovasc Med. 2025 Jul 18;12:1549377. doi: 10.3389/fcvm.2025.1549377 (PMC12313652; doi:10.3389/fcvm.2025.1549377)
Supplement: Supplementary file 7 [file Table2.docx]

Supplemental Table 2: Antibodies used for flow cytometry immunophenotyping

| **Specificity** | **Clone** | **Fluorochrome** | **Vendor** | **Dilution** | **Time** | **Purpose** |
| --- | --- | --- | --- | --- | --- | --- |
| CD45 | K252.1E4 | Pacific Blue | BioRad | Neat | 30’ | Lymphocyte lineage |
| CD3 | BB23-8E6-8C8 | PerCP-Cy5.5 | BD Biosciences | Neat | 30’ | T cell lineage |
| CD4 | 74-12-4 | PE-Cy7 | BD Biosciences | Neat | 30’ | Helper T cell |
| CD8 | 11/295/33 | Alexa Fluor 647 | BioRad | Neat | 30’ | Cytotoxic T cell |
| CD21 | B-ly4 | BV510 | BD Biosciences | Neat | 30’ | B cell |
| CD25 | K231.3B2 | Alexa Fluor 488 | BioRad | Neat | 30’ | Regulatory T cell |
| CD56 | MEM-188 | PE | BioLegend | Neat | 30’ | NK cell lineage |
| LIVE/DEAD |  | Near-IR | ThermoFisher Scientific | 1:2000 | 30’ | Viability |
