# Supplementary material for: A porcine model of acute rejection for cardiac transplantation
Source: Front Cardiovasc Med. 2025 Jul 18;12:1549377. doi: 10.3389/fcvm.2025.1549377 (PMC12313652; doi:10.3389/fcvm.2025.1549377)
Supplement: Supplementary file 8 [file Table3.docx]

Supplemental Table 3: Lymphocyte identification strategy

| **Subset** | **Phenotype** |
| --- | --- |
| Helper T cells | CD45^+^CD3^+^CD21^-^CD4^+^ |
| Cytotoxic T cells | CD45^+^CD3^+^CD21^-^CD8^+^ |
| Regulatory T cells | CD45^+^CD3^+^CD21^-^CD4^+^CD8^-^CD25^+^ |
| B cells | CD45^+^CD3^-^CD21^+^ |
| NK cells | CD45^+^CD21^-^CD56^+^ |
